# *Naja atra* SVPLA_2_ Aggravates Acute Kidney Injury Through Metabolic Reprogramming-Dependent Macrophage Polarization and Defective Efferocytosis

**DOI:** 10.3390/toxins18040155

**Published:** 2026-03-24

**Authors:** Jiahao Liu, Zejing Wen, Sunkun Tang, Jiajia Wu, Xiaowen Bi, Yang Yang, Chunhong Huang

**Affiliations:** 1 Health Management Center, The Second Affiliated Hospital of Nanchang University, Jiangxi Medical College, Nanchang University, Nanchang 330006, China; 2 Department of Biochemistry and Molecular Biology, School of Basic Medical Sciences, Jiangxi Medical College, Nanchang University, Nanchang 330006, China; 3 The First School of Clinical Medicine, Jiangxi Medical College, Nanchang University, Nanchang 330006, China

**Keywords:** *Naja atra*, SVPLA_2_, acute kidney injury, macrophage polarization, immunometabolism, efferocytosis

## Abstract

Snakebite envenoming remains a major global health challenge. *Naja atra* (*N. atra*) envenomation induces severe acute kidney injury (AKI), largely driven by snake venom phospholipase A_2_ (SVPLA_2_). Increasing evidence suggests that immune dysregulation, in addition to direct cytotoxicity, contributes to delayed renal injury. Here, we investigated whether *N. atra* SVPLA_2_ exposure is associated with macrophage immunometabolic remodeling and functional changes relevant to AKI progression. In vivo, AKI was induced in C57BL/6J mice by intraperitoneal administration of *N. atra* venom, followed by treatment with the SVPLA_2_ inhibitor varespladib. In vitro, bone marrow–derived macrophages were exposed to venom with or without varespladib. *N. atra* venom exposure was associated with extensive tubular apoptosis, increased renal macrophage abundance, and elevated kidney injury biomarkers. Macrophages exhibited a shift toward a pro-inflammatory polarization signature accompanied by reduced efferocytic capacity. Targeted metabolomics revealed coordinated increases in glycolytic intermediates together with upregulation of key glycolytic enzymes. Pharmacological inhibition of SVPLA_2_ partially restored macrophage metabolic features and efferocytic capacity and was accompanied by attenuation of renal injury. Together, these findings support a model in which SVPLA_2_ exposure is associated with macrophage immunometabolic remodeling and impaired apoptotic cell clearance during venom-induced AKI.

## 1. Introduction

Snakebite envenoming, recognized by the World Health Organization (WHO) as a priority neglected tropical disease, imposes a substantial global health burden, with an estimated 2.7 million cases annually leading to over 100,000 deaths and 400,000 permanent disabilities [1,2,3,4,5].

In Southern China and Southeast Asia, *Naja atra* (*N. atra*) envenomation constitutes a critical medical emergency. Beyond the immediate lethality, envenoming frequently causes severe local tissue destruction and delayed organ dysfunction that are difficult to reverse once established. Acute kidney injury (AKI) is one of the most common complications, which significantly contributes to morbidity and mortality despite antivenom administration [6,7]. The venom’s major toxic component, snake venom phospholipase A_2_ (SVPLA_2_), is a potent inducer of local tissue damage, hemolysis, and a profound, dysregulated systemic inflammatory response [8,9,10]. While the specific phospholipase A_2_ (PLA_2_) inhibitor varespladib has shown promise in neutralizing these effects clinically, the precise molecular cascades through which SVPLA_2_ drives distal organ injury, particularly AKI, remain incompletely defined [11,12,13]. This gap hinders the development of targeted adjunctive therapies aimed at mitigating the pathological sequelae that antivenom alone cannot promptly reverse.

Accumulating evidence emphasize the importance of investigating the pathogenesis of AKI from a comprehensive perspective [14,15,16]. In this context, the development of AKI extends beyond direct cytolytic injury to involve dysregulated immune responses, in which macrophages—as central orchestrators of innate immunity and tissue homeostasis—play a decisive role [17,18]. Previous studies have reported that snake venoms can stimulate macrophage activation characterized by increased inflammatory mediator production and altered phagocytic activity. However, these observations have largely focused on generalized inflammatory outputs or bulk phagocytosis, leaving the mechanistic pathways linking venom exposure to macrophage functional reprogramming during distal organ injury incompletely understood. In particular, whether venom-derived toxins directly reshape macrophage immunometabolism in a way that compromises their reparative functions within injured organs remains unclear.

A hallmark of *N. atra* SVPLA_2_-induced renal injury is the conspicuous accumulation of apoptotic tubular epithelial cells. Under physiological conditions, these apoptotic cells are rapidly cleared through efferocytosis, a specialized form of phagocytosis mediated primarily by tissue macrophages. Unlike general phagocytosis, which broadly refers to the uptake of pathogens or particles, efferocytosis specifically governs the recognition and removal of apoptotic cells, thereby preventing secondary necrosis and limiting the amplification of inflammatory signaling. Efficient efferocytosis is therefore not merely a waste-disposal mechanism but an active anti-inflammatory process essential for maintaining tissue homeostasis and promoting injury resolution [19,20,21]. In the context of toxin-induced AKI, where extensive tubular apoptosis occurs, impaired efferocytosis could allow apoptotic debris to accumulate and undergo secondary necrosis, releasing danger-associated molecular patterns that exacerbate inflammation and hinder renal repair. Intriguingly, our preliminary observations revealed that SVPLA_2_-intoxicated kidneys display robust macrophage infiltration accompanied by persistent apoptotic debris, suggesting a potential defect in this critical clearance pathway. This presents a pathophysiological paradox: macrophages are recruited to the site of injury yet appear unable to efficiently execute their reparative duties. Such dysfunction may arise from a fundamental reprogramming of macrophage phenotype and function [22].

Macrophage plasticity is central to injury resolution. Classically activated (M1) macrophages, which propagate inflammation and exacerbate tissue damage, are metabolically wired to rely on aerobic glycolysis. In contrast, alternatively activated (M2) macrophages, which promote repair and execute efferocytosis, predominantly utilize oxidative phosphorylation and fatty acid oxidation (FAO) [23,24,25]. This intrinsic link between cellular metabolism and functional fate suggests that disrupting metabolic pathways could forcibly skew macrophage polarization, locking them into a pro-damage state [26]. We thus hypothesize that SVPLA_2_ instigates a pathogenic metabolic switch in renal macrophages, driving them toward a glycolytically dependent, pro-inflammatory M1 state while impairing their essential efferocytic capacity, thereby perpetuating *N. atra* triggered AKI. Therefore, to investigate this, we employed a multi-tiered experimental strategy. First, we established the in vivo landscape, confirming *N. atra* SVPLA_2_-induced AKI and dissecting the concurrent dynamics of apoptosis, macrophage infiltration, and polarization within the kidney. Then, we utilized bone marrow-derived macrophages (BMDMs) to mechanistically probe the direct effects of SVPLA_2_ on polarization, efferocytosis, and central carbon metabolism.

Taken together, our integrated analysis reveals that SVPLA_2_ triggers a profound glycolytic reprogramming, characterized by the upregulation of key rate-limiting enzymes, which drives M1 polarization and functionally cripples efferocytosis. This work shifts the paradigm of SVPLA_2_ toxicity from one of passive cellular necrosis to an active immunometabolic sabotage of tissue repair mechanisms. By establishing a direct link between snake venom exposure, macrophage metabolic reprogramming, and defective efferocytosis, our study provides a new conceptual framework for investigating immune-mediated mechanisms of venom-induced organ injury.

## 2. Results

### 2.1. N. atra SVPLA_2_ Induces Acute Kidney Injury in Mice

**Figure 1:**
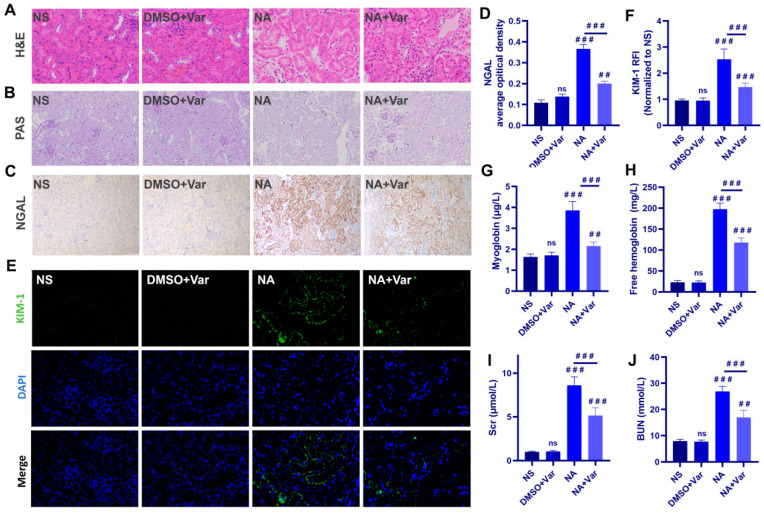
*N. atra* SVPLA_2_ induces AKI in mice. (**A**,**B**) Representative H&E and PAS staining of kidney sections. (**C**) Representative immunohistochemistry staining of NGAL. (**D**) Quantification of NGAL average optical density. (**E**) Representative immunofluorescence staining of KIM-1 (green) with DAPI (blue). (**F**) Relative fluorescence intensity (RFI) of KIM-1 normalized to NS group. (**G**,**H**) Serum myoglobin and free hemoglobin levels. (**I**,**J**) SCr and BUN levels. For NA group, mice were administered *N. atra* venom at 0.5 LD_50_ (27.5 mg/kg). For NA + Var group, mice received the same dose of *N. atra* venom combined with varespladib (1.75 mg/kg). Mice were euthanized and tissues were collected for analysis 12 h after venom injection. Data are presented as mean ± SD. ^##^ *p* < 0.01, and ^###^ *p* < 0.001. ns, not significant vs. indicated group. *n* = 5 (in vivo), biological replicates.

Histopathological analysis revealed that *N. atra* venom (NA) induced marked renal tubular injury, characterized by tubular dilation, increased renal interstitium, epithelial disruption, and brush border loss on Hematoxylin and Eosin (H&E) and Periodic Acid–Schiff (PAS) staining; these changes were substantially alleviated by varespladib treatment (Figure 1A,B). PAS staining further indicated abnormal glycogen depletion in the NA group. Consistent with the structural damage, expression of the acute kidney injury biomarkers neutrophil gelatinase-associated lipocalin (NGAL) and kidney injury molecule-1 (KIM-1) was significantly upregulated [27]. Immunohistochemistry showed strong NGAL induction in renal tubules after venom exposure (Figure 1C,D), while immunofluorescence confirmed a robust increase in KIM-1, which was significantly attenuated by varespladib (Figure 1E,F).

Systemic biomarkers confirmed severe rhabdomyolysis and hemolysis in the NA group, evidenced by marked elevations in serum myoglobin and free hemoglobin (Figure 1G,H). Consequently, renal function was significantly impaired, with sharp increases in serum levels of creatinine (SCr) and blood urea nitrogen (BUN). All these venom-induced alterations were significantly reduced by varespladib co-treatment (Figure 1I,J). Taken together, the aforementioned results indicate that 0.5 LD_50_ (27.5 mg/kg) of *N. atra* venom can induce AKI in mice, and administering 1.75 mg/kg varespladib to inhibit SVPLA_2_ in *N. atra* venom can effectively alleviate these injuries.

### 2.2. N. atra SVPLA_2_ Induces Widespread Renal Apoptosis Without Reducing Macrophage Abundance

**Figure 2:**
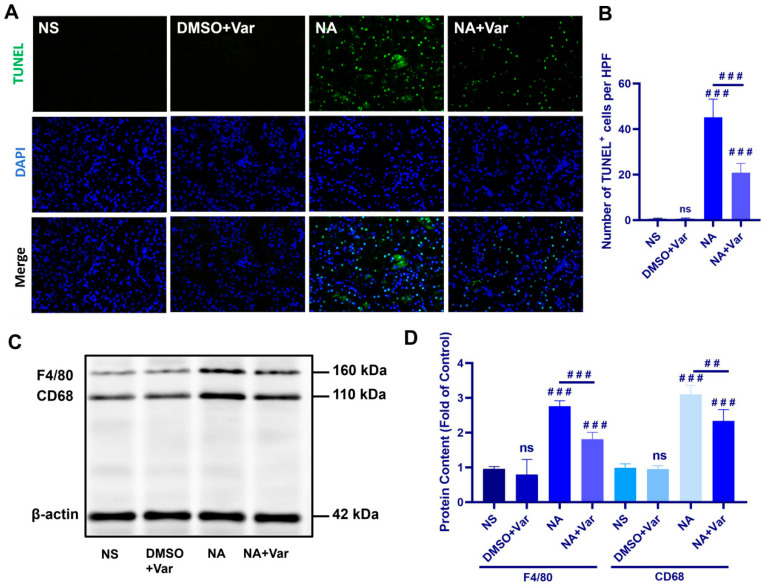
SVPLA_2_ induces renal apoptosis without reducing macrophage abundance. (**A**) Representative TUNEL staining of kidney sections. (**B**) Quantification of TUNEL-positive cells per high-power field. (**C**) Representative Western blot of F4/80 and CD68 in renal tissues. (**D**) Densitometric analysis of F4/80 and CD68 protein levels normalized to β-actin. For NA group, mice were administered *N. atra* venom at 0.5 LD_50_ (27.5 mg/kg). For NA + Var group, mice received the same dose of *N. atra* venom combined with varespladib (1.75 mg/kg). Mice were euthanized and tissues were collected for analysis 12 h after venom injection. Data are presented as mean ± SD. ^##^ *p* < 0.01, and ^###^ *p* < 0.001. ns, not significant vs. indicated group. *n* = 5 (in vivo), biological replicates.

TUNEL staining revealed a marked increase in apoptotic cells in the kidneys of *N. atra* venom–treated mice compared with NS and DMSO + Var controls, whereas varespladib treatment significantly reduced the number of TUNEL-positive cells (Figure 2A,B).

Under physiological conditions, apoptotic cells are rapidly recognized and cleared by surrounding phagocytes, particularly macrophages, thereby preventing the accumulation of cellular debris and subsequent inflammatory responses. Contrary to this hypothesis, Western blot analysis revealed that venom treatment did not impair, but rather significantly enhanced, macrophage abundance within injured renal tissue was evaluated using the commonly used macrophage markers F4/80 and CD68. Notably, these markers allow assessment of the overall macrophage population but do not distinguish between resident and infiltrating macrophage subsets (Figure 2C,D). These findings indicate that SVPLA_2_ induces extensive renal apoptosis despite a concurrent increase in renal macrophage abundance, suggesting that apoptotic cell clearance may be functionally insufficient at this stage of injury.

### 2.3. N. atra SVPLA_2_ Induces a Pro-Inflammatory Polarization Signature in Renal Macrophages

Given that *N. atra* venom exposure did not reduce total renal macrophage abundance, yet resulted in a pronounced accumulation of apoptotic cells, we hypothesized that impaired macrophage efferocytosis rather than defective macrophage recruitment accounted for this phenomenon. Because efficient efferocytosis is commonly associated with macrophage states enriched in reparative or M2-like functional programs, we next investigated whether SVPLA_2_ exposure alters macrophage polarization signatures in the kidney in vivo.

**Figure 3:**
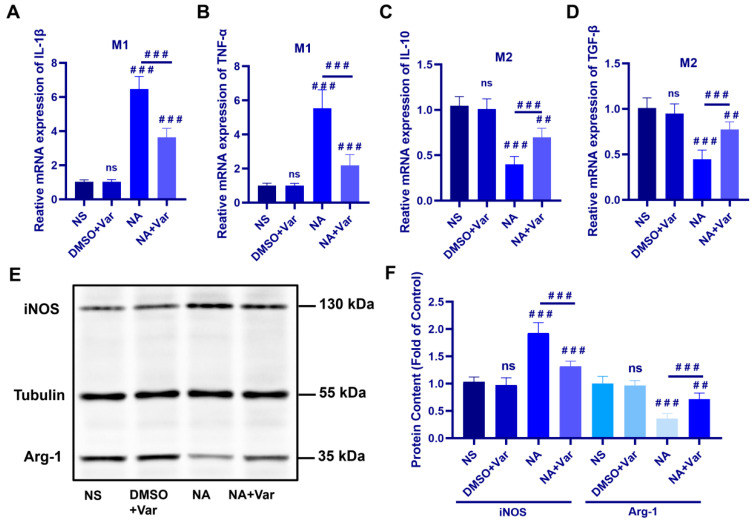
SVPLA_2_ drives M1 polarization and suppresses M2 polarization of renal resident macrophages. (**A**–**D**) Relative mRNA expression of M1 markers (*Il1b*, *Tnfα*) and M2 markers (*Il10*, *Tgfb*) in renal tissues. (**E**) Representative Western blot showing iNOS and Arg-1 protein expression in renal tissues. (**F**) Densitometric quantification of iNOS and Arg-1 normalized to Tubulin. For NA group, mice were administered *N. atra* venom at 0.5 LD_50_ (27.5 mg/kg). For NA + Var group, mice received the same dose of *N. atra* venom combined with varespladib (1.75 mg/kg). Mice were euthanized and tissues were collected for analysis 12 h after venom injection. Data are presented as mean ± SD. ^##^ *p* < 0.01, and ^###^ *p* < 0.001. ns, not significant vs. indicated group. *n* = 5 (in vivo), biological replicates.

Quantitative Real-Time Polymerase Chain Reaction (RT-qPCR) analysis showed that *N. atra* venom exposure markedly upregulated the mRNA levels of the pro-inflammatory M1 markers *Il1b* and *Tnfα* compared with control groups, whereas varespladib treatment significantly attenuated this induction (Figure 3A,B). In contrast, the expression of the anti-inflammatory M2 markers *Il10* and *Tgfb* was significantly reduced following venom administration and partially restored by varespladib (Figure 3C,D).

Consistent with the transcriptional changes, Western blot analysis revealed a pronounced increase in inducible nitric oxide synthase (iNOS) (M1 marker) protein levels accompanied by a concomitant decrease in arginase 1 (Arg-1) (M2 marker) expression in the kidneys of venom-treated mice (Figure 3E,F). These alterations were significantly reversed upon SVPLA_2_ inhibition. Collectively, at the selected experimental time point, these results indicate that SVPLA_2_ skews renal resident macrophages toward an M1-dominant phenotype while suppressing M2 polarization in vivo.

### 2.4. N. atra SVPLA_2_ Induces a Pro-Inflammatory Polarization Signature and Reduces Efferocytic Capacity in BMDMs

To determine whether SVPLA_2_ directly influences macrophage functional programs independent of the renal microenvironment, BMDMs were exposed to *N. atra* venom in vitro.

**Figure 4:**
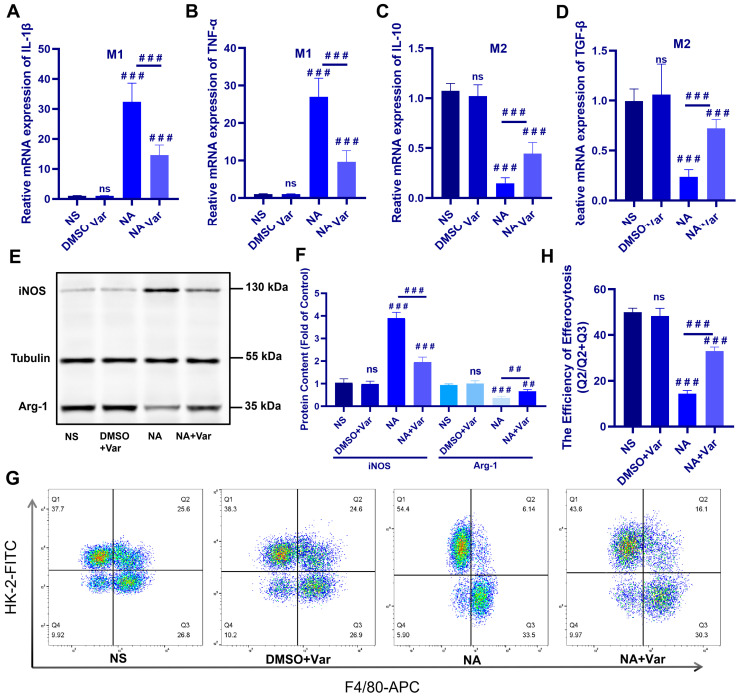
SVPLA_2_ promotes M1 polarization and impairs efferocytosis in BMDMs in vitro. (**A**–**D**) Relative mRNA expression of M1 markers (*Il1b*, *Tnfα*) and M2 markers (*Il10*, *Tgfb*) in BMDMs. (**E**) Representative Western blot showing iNOS and Arg-1 expression in BMDMs. (**F**) Densitometric quantification of iNOS and Arg-1 normalized to Tubulin. (**G**) Representative flow cytometric plots showing efferocytosis of apoptotic HK-2 cells by F4/80^+^ macrophages. (**H**) Quantification of efferocytotic efficiency. For the NA group, BMDMs were treated with *N. atra* venom at 4 µg/mL. For the NA + Var group, BMDMs received the same concentration of *N. atra* venom combined with varespladib (3 µM). BMDMs were analyzed 24 h after treatment. Data are presented as mean ± SD. ^##^ *p* < 0.01, and ^###^ *p* < 0.001. ns, not significant vs. indicated group. *n* = 3 (in vitro), biological replicates.

Consistent with the in vivo observations, SVPLA_2_ exposure induced a pro-inflammatory polarization signature in BMDMs. This was reflected by increased expression of the pro-inflammatory markers *Il1b*, *Tnfα*, and *inos*, together with reduced expression of genes commonly associated with reparative macrophage programs, including *Il10*, *Tgfb*, and *Arg1* (Figure 4A–F). Co-treatment with the specific PLA_2_ inhibitor varespladib markedly attenuated these changes, supporting the specificity of the response to SVPLA_2_ activity. Together, these data indicate that SVPLA_2_ exposure is associated with a shift toward a pro-inflammatory macrophage functional program in vitro.

Because efficient efferocytosis is commonly associated with macrophage states enriched in reparative or M2-like functional programs, we next examined whether SVPLA_2_ affects macrophage-mediated clearance of apoptotic cells. Flow cytometric analysis revealed a marked reduction in the uptake of apoptotic HK-2 cells by SVPLA_2_-treated macrophages compared with controls, whereas varespladib treatment significantly restored efferocytic capacity (Figure 4G,H).

Collectively, these findings show that SVPLA_2_ exposure promotes a pro-inflammatory polarization signature in BMDMs and is accompanied by reduced macrophage efferocytic capacity in vitro. This observation may provide a potential mechanistic explanation for the accumulation of apoptotic cells observed in vivo following *N. atra* venom exposure despite increased macrophage abundance in the kidney.

### 2.5. Central Carbon Metabolomics Reveals Glycolytic Metabolic Remodeling in SVPLA_2_-Treated Macrophages

Macrophage polarization is tightly coupled to cellular metabolism [22,23,24,25,26]. Given the venom-associated shift toward a pro-inflammatory macrophage program, we investigated whether *N. atra* SVPLA_2_ exposure alters the central carbon metabolism of macrophages. We performed targeted metabolomics profiling, which quantified 41 key metabolites across four core metabolic modules: (i) glycolysis, (ii) the tricarboxylic acid (TCA) cycle, (iii) the pentose phosphate pathway (PPP), (iv) energy & redox cofactors.

**Figure 5:**
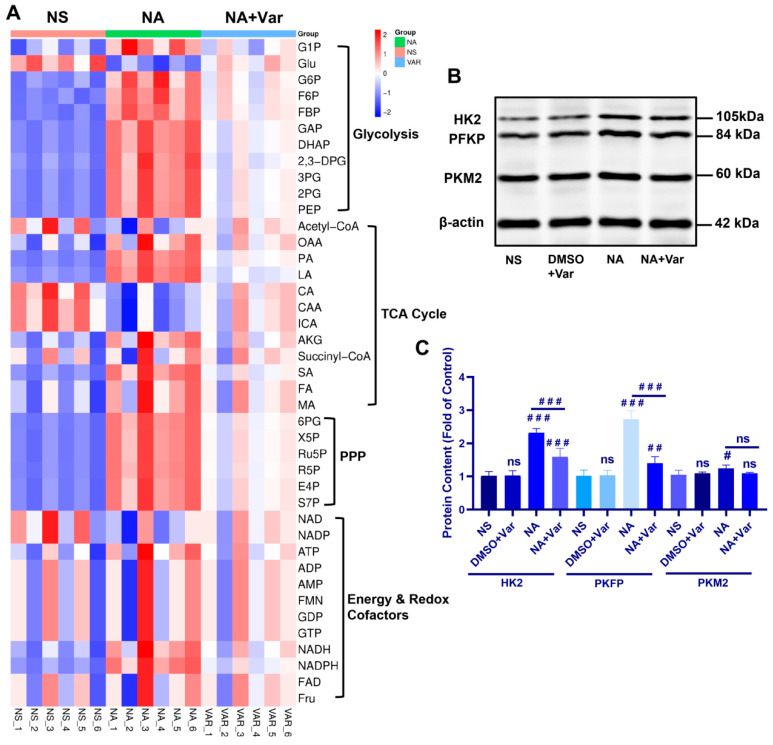
Central carbon metabolomics identifies SVPLA_2_-driven glycolytic reprogramming in macrophages. (**A**) Heatmap of targeted central carbon metabolites in BMDMs. (**B**) Representative Western blot of HK2, PFKP, and PKM2 in BMDMs. (**C**) Densitometric quantification of HK2, PFKP, and PKM2 normalized to β-actin. For the NA group, BMDMs were treated with *N. atra* venom at 4 µg/mL. For the NA + Var group, BMDMs received the same concentration of *N. atra* venom combined with varespladib (3 µM). BMDMs were analyzed 24 h after treatment. Data are presented as mean ± SD. ^#^ *p* < 0.05, ^##^ *p* < 0.01, and ^###^ *p* < 0.001. ns, not significant vs. indicated group. *n* = 3 (in vitro), biological replicates.

Analysis revealed a distinct metabolic pattern, with SVPLA_2_-treated macrophages (NA group, green) exhibiting coordinated increases in multiple glycolytic intermediates. This included upstream metabolites such as glucose-6-phosphate (G6P) and fructose-6-phosphate (F6P), the committed metabolite fructose-1,6-bisphosphate (F1,6BP), and downstream products phosphoenolpyruvate (PEP) and lactate (Figure 5A). In contrast, changes in the TCA cycle and PPP were less pronounced and pathway-wide. Notably, varespladib co-treatment largely normalized this venom-induced glycolytic signature.

To establish a mechanistic link between the observed metabolic rewiring and enzyme expression, we examined the protein levels of three key rate-limiting glycolytic enzymes: hexokinase 2 (HK2, catalyzing the first committed step), phosphofructokinase platelet type (PFKP, the major flux-controlling enzyme), and pyruvate kinase M2 isoform (PKM2, the final regulatory node). Consistent with the accumulation of upstream glycolytic metabolites, SVPLA_2_ exposure significantly increased the protein abundance of both HK2 and PFKP, effects that were attenuated by varespladib (Figure 5B,C). Interestingly, PKM2 protein levels remained unchanged. This observation is consistent with previous reports showing that PKM2 in macrophages is often regulated through post-translational mechanisms—such as changes in oligomerization state or phosphorylation—rather than through changes in total protein abundance [26]. Therefore, the observed accumulation of glycolytic intermediates may reflect enhanced upstream control through HK2 and PFKP. This distinct pattern—upregulation of early and mid-pathway gatekeepers (HK2, PFKP) with stable terminal enzyme (PKM2) expression—aligns with a model wherein SVPLA_2_ enhances glycolytic flux and concurrently may promote the metabolic branching typical of M1 macrophages, potentially facilitating the diversion of glycolytic intermediates into biosynthetic pathways commonly associated with inflammatory macrophage activation. Taken together, these data indicate that SVPLA_2_ exposure is associated with a glycolysis-biased metabolic program in macrophages, which is partially reversible by pharmacologic PLA_2_ inhibition.

## 3. Discussion

Owing to the scarcity of adjunct targeted interventions for organ injury and the incomplete understanding of venom toxicity mechanisms, organ dysfunction associated with snakebite envenoming remains a major global health challenge [1,2,3,4,5]. Among these, AKI is the most frequent and clinically consequential complications. Although antivenom and emerging toxin-directed inhibitors can mitigate early toxicity, delayed renal injury often persists, implying that secondary pathobiology contributes to disease progression. In this context, defining how venom components reshape host immunity and repair programs is essential for developing adjunct strategies beyond neutralization [11]. Here, we demonstrate that *N. atra* SVPLA_2_ drives a pro-inflammatory immunometabolic switch in macrophages. Specifically, it enhances glycolytic flux, promotes M1 polarization while suppressing M2 polarization, and critically impairs efferocytic function. This study moves beyond the traditional toxicological focus on the direct actions of toxins and suggests a potential immunometabolic framework through which venom components may influence macrophage function during venom-induced organ injury.

Although no studies have directly reported the effects of *N. atra* venom SVPLA_2_ on macrophage polarization or efferocytosis, numerous studies have documented the connection between other snake venoms and macrophages. For instance, a paradoxical macrophage state has been repeatedly described in studies of *Crotalus durissus terrificus* venom and crotoxin: macrophages exhibit increased effector outputs including oxidative and nitrogen reactive species and antimicrobial activity, while showing reduced spreading and phagocytic capacity [28,29]. Importantly, these inhibitory effects on phagocytosis occur rapidly in vitro and in vivo and are attributable to venom toxins. Metabolic analyses further indicate that venom exposure can enhance glycolysis and glutaminolysis alongside this functional impairment [29]. Together, these observations provide a conceptual basis for interpreting venom as an immunomodulatory stimulus that pushes macrophages into an inflammatory, yet clearance-defective state.

Our kidney data align with this “activated but dysfunctional” paradigm. Despite increased renal macrophage abundance after *N. atra* envenomation, apoptotic tubular cells accumulated markedly, indicating that macrophage recruitment alone is insufficient for injury resolution. This mismatch may indicate a functional defect in apoptotic cell clearance—efferocytosis—rather than a failure of macrophage presence. While efferocytosis shares core machinery with phagocytosis, it is functionally specialized for silent removal of apoptotic cells and is typically associated with reparative macrophage programs [30]. Therefore, sustained apoptotic debris in the setting of macrophage infiltration strongly points to venom-imposed impairment of pro-resolving macrophage functions.

Since efficient efferocytosis is commonly associated with macrophage states enriched in reparative or M2-like functional programs, we hypothesized that snake venom might impair this process by shifting macrophage polarization toward a more pro-inflammatory state. It should be noted that macrophage activation in vivo represents a continuum of states rather than a strict M1/M2 dichotomy; therefore, the terminology used here serves primarily as a functional framework to describe polarization trends [20]. Our in vivo experiments confirmed that the venom increases the expression of the M1 macrophage markers while reducing the levels of M2 markers. Notably, BMDMs differentiated with M-CSF often display a homeostatic/reparative-leaning baseline phenotype (M2-biased baseline). Consequently, even under untreated conditions, they show higher Arg-1 expression and greater efferocytic efficiency, both of which are significantly reduced after venom treatment—consistent with the in vivo findings. Thus, our study establishes a novel linking SVPLA_2_ with macrophage efferocytosis, elucidating the mechanism behind the substantial accumulation of apoptotic cells in the kidney induced by SVPLA_2_.

Pro-inflammatory macrophage activation has been implicated in venom-induced inflammatory pathology. For example, our observed M1-dominant polarization aligns with extensive evidence that venom-derived sPLA_2_s activate canonical inflammatory signaling in macrophages. An Asp49 sPLA_2_ has been shown to induce COX-2 expression and PGE2 production through NF-κB activation and upstream p38MAPK and PKC signaling [31]. Related work comparing distinct venom sPLA_2_s demonstrates that toxins can differentially engage COX-1/COX-2 regulation and arachidonic acid release through distinct phospholipase dependencies, underscoring that venom sPLA_2_s encode diverse inflammatory “programs” rather than a single stereotyped response. Whole venom exposure can similarly promote COX-2 upregulation and prostaglandin production in leukocytes and in isolated macrophages and neutrophils, supporting macrophages as direct cellular targets during envenomation [32]. In human macrophage models, venom components can also induce inflammatory cytokines and chemokines, further illustrating broad activation of inflammatory mediator networks [33]. Within this established inflammatory framework, our results extend the narrative by showing that SVPLA_2_ not only activates inflammatory outputs but also reshapes macrophage fate decisions toward an M1-like state while suppressing reparative features.

Macrophage polarization is highly plastic and sensitive to dose and microenvironmental context, and macrophage populations in injured tissues exist along a heterogeneous activation spectrum rather than discrete M1/M2 subsets [34,35,36]. Another key advance of our study is the mechanistic coupling between macrophage phenotype and metabolic state. Prior work with crotalid venom demonstrated increased activities of glycolytic and glutaminolytic enzymes in macrophages alongside reduced phagocytic activity, consistent with the idea that venom can reconfigure macrophage metabolism during functional reprogramming [29]. Metabolomics is recognized as an emerging tool for studying snake venom toxicology, offering novel perspectives into the mechanisms of snake venom toxicity [37,38,39]. In this study, our targeted metabolomics revealed coordinated accumulation of glycolytic intermediates and selective upregulation of HK2 and PFKP in SVPLA_2_-treated macrophages, indicating a glycolysis-biased metabolic configuration. Such a metabolic shift not only sustains pro-inflammatory transcriptional programs but also likely creates a metabolic environment incompatible with the energetic and biosynthetic demands of pro-resolving functions, such as efficient efferocytosis. In this way, immunometabolic rewiring may provide a conceptual link connecting SVPLA_2_ exposure to persistent inflammation and defective clearance of apoptotic cells. However, a limitation of the present study is that mitochondrial respiration and oxidative metabolic pathways were not directly assessed. Although the current metabolomics and enzyme analyses indicate enhanced glycolytic activity, future studies examining mitochondrial function and oxidative metabolism will be necessary to more comprehensively define the immunometabolic remodeling of macrophages following SVPLA_2_ exposure.

Additionally, varespladib not only attenuated renal injury but also reversed macrophage polarization imbalance, normalized glycolytic reprogramming, and restored efferocytotic capacity. These coordinated rescues highlight that venom-induced immunometabolic dysfunction is pharmacologically reversible and suggest that early SVPLA_2_ inhibition may complement antivenom by preventing a downstream self-amplifying loop of inflammation and defective clearance. Conceptually, this reframes toxin inhibition as both neutralization and immunometabolic correction.

Several limitations of the present study should be noted. First, we did not distinguish resident versus monocyte-derived renal macrophage subsets, which may differ in metabolic wiring and efferocytosis competence. Second, while our data strongly associate glycolytic reprogramming with efferocytosis impairment, causal testing using macrophage-specific metabolic interventions will be needed. Future work integrating cell subset tracing and mechanistic metabolic perturbation should further refine this framework and strengthen its translational relevance.

The present study did not employ purified SVPLA_2_ to investigate its toxic mechanisms. Instead, SVPLA_2_ activity was selectively inhibited within crude *N. atra* venom using varespladib. This approach was adopted because accumulating evidence indicates that venom toxicity arises from synergistic interactions among multiple toxin components, and the use of isolated purified toxins may not fully recapitulate their functional contributions within the native venom context. Accordingly, this experimental design was chosen to more faithfully reproduce the pathophysiological effects of *N. atra* envenomation in vivo, as previously reported [8]. Therefore, the results of this study only confirm that varespladib can partially neutralize the toxic effects of *N. atra* venom by inhibiting SVPLA_2_, but it cannot completely reverse the effects. Thus, the action of other toxins should not be overlooked.

Another limitation of the present study is that the in vivo experiments were performed using a single SVPLA_2_ dose and a single post-exposure time point. Given that macrophage polarization and immunometabolic remodeling are highly dynamic processes, the current results should be interpreted as representing a defined stage of established renal injury rather than the full temporal evolution of venom-induced AKI. Future studies incorporating multiple early and late time points, as well as dose-ranging designs, will be valuable for delineating the kinetics and stage specificity of macrophage functional changes following envenomation. Furthermore, this study shows that macrophage abundance was evaluated using F4/80 and CD68 staining, which does not allow for definitive discrimination between resident kidney macrophages and infiltrating monocyte-derived macrophages. These populations may exhibit distinct functional properties, including differences in efferocytosis and inflammatory responses. Future studies incorporating additional subset markers or lineage-tracing approaches will help clarify the relative contributions of these macrophage populations during *N. atra* SVPLA_2_-induced renal injury.

## 4. Conclusions

These findings show that *N. atra* SVPLA_2_ exposure is associated with a macrophage immunometabolic shift during venom-induced AKI. SVPLA_2_ treatment enhances glycolytic metabolic signatures and biases macrophages toward a pro-inflammatory functional program while impairing efferocytic capacity, which may contribute to the accumulation of apoptotic cells and sustained renal inflammation. Importantly, pharmacological inhibition of SVPLA_2_ partially restores macrophage metabolic balance and efferocytic function and is accompanied by reduced renal injury. Together, these findings support a model in which venom-derived toxins influence macrophage immunometabolism and clearance functions, thereby shaping the inflammatory microenvironment during venom-induced organ injury. This work provides a conceptual framework for understanding how venom components may contribute to organ pathology through immunometabolic mechanisms.

## 5. Methods

### 5.1. Main Reagents

Detailed information on the main reagents, primary antibodies, and secondary antibodies are provided in Table S1, Table S2 and Table S3, respectively, in Supplementary Materials.

### 5.2. Animal Ethics and Animal Experimental Design

All animal experimental protocols were approved by the Institutional Animal Care and Use Committee of Nanchang University (Ethics Code: NCULAE-20220624042). Male C57BL/6J mice (7–8 weeks old, 30 ± 5 g) were obtained from the Nanchang University Animal Center and randomly assigned into four groups (*n* = 5 per group): (i) normal saline control (NS), (ii) DMSO + varespladib vehicle control (DMSO + Var), (iii) *N. atra* venom only (NA), and (iv) *N. atra* venom followed by varespladib treatment at 15 min post-envenomation to simulate clinical snakebites (NA + Var).

*N. atra* venom was administered via intraperitoneal injection at a dose of 0.5 LD_50_ (27.5 mg/kg), which can induce AKI in mice based on previous literature [11]. Varespladib was dissolved in DMSO to prepare a 10 mg/mL stock solution and stored at −20 °C. For in vivo administration, the stock solution was diluted with normal saline to a final working concentration of 50 μg/mL and administered intraperitoneally at a dose of 1.75 mg/kg, based on previous studies [8,40].

The total injection volume was adjusted to 150 μL with normal saline for all treatments. Mice were euthanized 12 h post-injection. Prior to tissue collection, mice were deeply anesthetized via inhalation of a gradually increased concentration of CO_2_. Once a surgical plane of anesthesia was confirmed by the absence of pedal reflex, euthanasia was completed by exsanguination via cardiac puncture. EDTA-anticoagulated blood samples were collected during this procedure. Subsequently, kidneys were harvested. One portion of kidney tissue was collected for follow-up analysis.

### 5.3. Isolation and Differentiation of BMDMs and Viability Assay

Bone marrow cells were harvested from the femurs and tibiae of C57BL/6J mice by flushing the marrow cavities with PBS. After erythrocyte lysis using ACK lysis buffer, the isolated bone marrow cells were seeded in culture dishes at a density of 1 × 10^7^ cells per dish and differentiated in complete DMEM medium supplemented with 12% FBS, 1% penicillin-streptomycin, and 25 ng/mL recombinant mouse M-CSF. The medium was refreshed on day 3, and cells were cultured for a total of 7 days to allow for full differentiation into BMDMs.

Cell viability was assessed using the Cell Counting Kit-8 (CCK-8) assay (Abcam, Cambridge, UK). Cells were treated with increasing concentrations of *N. atra* venom (0, 0.5, 1, 2, 4, 8, and 10 µg/mL) for 24 h. Based on the experimental objective of this study, a venom concentration that maintained 80–90% cell viability while robustly inducing M1 macrophage polarization was selected as the working concentration. To investigate the dose of varespladib in BMDMs cells, after establishing the venom working concentration, cells were treated with varespladib at graded concentrations (0, 0.3, 1, 3, and 10 µM). Cells were co-treated with varespladib and venom simultaneously for 24 h. A varespladib concentration that maintained 85–95% cell viability while significantly attenuating venom-induced M1 polarization was selected as the optimal intervention dose.

Ultimately, we determined that the effective concentration of *N. atra* venom for treating BMDMs was 4 µg/mL, which maintained cell viability at 88 ± 2% and significantly induced M1 polarization of BMDMs. The protective concentration of varespladib was 3 µM, which preserved cell viability at 94 ± 2% and markedly attenuated M1 polarization induced by 4 µg/mL *N. atra* venom in BMDMs.

### 5.4. Histopathology

Kidney tissues were fixed in 4% paraformaldehyde for 72 h, embedded in paraffin, and sectioned at a thickness of 5 μm. Sections were deparaffinized, rehydrated, and stained with H&E or PAS to evaluate renal histopathological alterations.

### 5.5. Immunofluorescence and Immunohistochemistry

For immunofluorescence staining, kidney tissue sections were blocked with 10% Lowlenthal serum for 1 h and incubated with primary antibodies at 4 °C overnight, followed by incubation with fluorescent secondary antibodies at 37 °C for 1 h (protected from light). Nuclei were counterstained with DAPI. Images were acquired using an LSM 910 confocal microscope (ZEISS, Auberkheim, Germany).

For immunohistochemistry staining, tissue sections were subjected to antigen retrieval and endogenous peroxidase blocking, followed by blocking with 10% Lowlenthal serum for 1 h. Sections were incubated with primary antibodies at 4 °C overnight and HRP-conjugated secondary antibodies at room temperature for 1 h. Signals were visualized using a DAB substrate kit, and nuclei were counterstained with hematoxylin. Images were acquired using a bright-field microscope (Nikon, Tokyo, Japan).

### 5.6. TUNEL

Apoptosis in kidney tissue sections were detected using a TUNEL assay kit (Elabscience Biotechnology Co., Ltd, Beijing, China). After deparaffinization and rehydration, sections were permeabilized with Proteinase K (15 μg/mL, 15 min). Sections were then incubated with the TUNEL reaction mixture for 1 h at 37 °C, followed by nuclear counterstaining with DAPI. Images were acquired using a fluorescence microscope (Nikon), and TUNEL-positive cells were quantified in multiple random fields per section.

### 5.7. Assessment of Renal Function Biomarkers

Serum was obtained by centrifugation at 3000× *g* for 20 min. Serum levels of SCr, BUN, myoglobin, and free hemoglobin were measured using Assay Kits (Nanjing Jiancheng Bioengineering Institute, Nanjing, China). All measurements were performed in duplicate using a microplate reader (SpectraMax^®^ iD3, Molecular Devices, Shanghai, China).

### 5.8. Quantitative Real-Time Polymerase Chain Reaction (RT-qPCR)

Total RNA was isolated from cells or tissues with TRIzol reagent. Reverse transcription was performed using 1 μg total RNA and a TransGen All-in-One First-Strand cDNA Synthesis Kit (TransGen Biotech, Beijing, China). RT-qPCR was carried out on a Bio-Rad CFX Connect Real-Time System with SYBR Green Premix. Relative mRNA expression was normalized to *Gapdh* and calculated by the 2^−ΔΔCt^. All primer sequences are included in Table S4 in the Supplementary Materials.

### 5.9. Western Blot

Protein was resolved by SDS-PAGE and transferred onto PVDF membranes. The membranes were blocked in 5% BSA for 2 h and subsequently incubated with the primary antibodies (4 °C, overnight). After washing, HRP-conjugated secondary antibodies were applied for 1 h. The signal was visualized using enhanced chemiluminescence reagents and documented using an imaging system. All raw Western blot full membranes in this study are provided in Figure S1 (Supplementary Materials).

### 5.10. Efferocytosis Assay

Apoptotic HK-2 cells were induced by ultraviolet irradiation and labeled with 5 μM carboxyfluorescein succinimidyl ester (CFSE). Labeled apoptotic cells were co-cultured with BMDMs at a ratio of 5:1 for 45 min at 37 °C. After incubation, non-internalized apoptotic cells were removed by extensive washing with PBS. Cells were collected by gentle scraping, fixed with pre-cooled methanol (−20 °C) for 10 min, and immunostained with a phycoerythrin-conjugated anti-F4/80 antibody to identify macrophages.

### 5.11. Targeted Metabolomic Analysis of BMDMs

A targeted metabolomics approach focusing on central carbon metabolism was employed to profile intracellular metabolites in BMDMs. Cells from three experimental groups (Control, *N. atra*-treated, and *N. atra* + varespladib-treated) were collected, washed with cold PBS, and immediately snap-frozen in liquid nitrogen (six independent biological replicates). Metabolite extraction and subsequent liquid chromatography–tandem mass spectrometry (LC–MS/MS) analysis were performed by Neora Biotechnology Co., Ltd. (Nanchang, China).

Briefly, cell pellets were homogenized in 800 μL of pre-chilled methanol/acetonitrile (1:1, *v*/*v*) and subjected to ultrasonication in an ice bath for 20 min. After centrifugation at 14,000× *g* for 20 min at 4 °C, supernatants were collected for analysis. Metabolite separation and detection were conducted using a QTRAP™ 6500 LC–MS/MS system (SCIEX). Metabolite abundances were normalized to cell number prior to comparative analysis. Differential metabolites were defined as those with a fold change >1.5 and an adjusted *p* < 0.05. Data visualization and clustering analyses were performed using Cluster 3.0 and Java Treeview software (v 3.0), based on the previously described method [41].

### 5.12. Statistical Analysis

All animal experiments included five independent biological replicates (*n* = 5), while cell-based experiments included three independent biological replicates (*n* = 3). Data are presented as mean ± SD. Data distribution was initially assessed using the Shapiro–Wilk normality test, and variance homogeneity was evaluated using the Brown–Forsythe test. For datasets meeting assumptions of normality and homoscedasticity, statistical comparisons among multiple groups were performed using one-way analysis of variance followed by Tukey’s post hoc test. When these assumptions were not met, non-parametric Kruskal–Wallis tests followed by Dunn’s multiple comparison procedure were applied. A threshold of *p* < 0.05 was considered statistically significant. All statistical analyses were performed using GraphPad Prism (v 9.0).

## Data Availability

The original contributions presented in this study are included in the article/Supplementary Material. Further inquiries can be directed to the corresponding author(s).

## Supplementary Materials

The following supporting information can be downloaded at: https://www.mdpi.com/article/10.3390/toxins18040155/s1, Table S1: Main reagents used in this study; Table S2: Primary antibodies used in WB, IF, IHC, and flow cytometry; Table S3. Secondary antibodies used in WB, IF, and IHC; Table S4. Primer sequences used for RT-qPCR; Figure S1 All uncropped WB membranes in this study.
