# Supplementary material for: Naja atra SVPLA2 Aggravates Acute Kidney Injury Through Metabolic Reprogramming-Dependent Macrophage Polarization and Defective Efferocytosis
Source: Toxins (Basel). 2026 Mar 24;18(4):155. doi: 10.3390/toxins18040155 (PMC13120190; doi:10.3390/toxins18040155)
Supplement: Supplementary file 1 [file toxins-18-00155-s001.zip › toxins-4174201-supplementary.pdf]

Supplementary File S1

Naja atra SVPLA$_2$ aggravates acute kidney injury through metabolic reprogramming–dependent macrophage polarization and defective efferocytosis

Table S1. Main reagents used in this study

Item Name	Source	Catalog No.
Lyophilized *Naja atra* venom	Huangshan Snake Hacienda	N/A
Varespladib (LY315920)	MedChemExpress Co.,Ltd.	HY-13402
One-step TUNEL In Situ Apoptosis Kit	Elabscience Biotechnology Co.,Ltd.	E-CK-A320
Cell Counting Kit 8	Abcam	ab228554
Scr Assay Kit	Nanjing Jiancheng Bioengineering Institute	C011-2-1
BUN Assay Kit	Nanjing Jiancheng Bioengineering Institute	C013-2-1
Myoglobin Assay Kit	Nanjing Jiancheng Bioengineering Institute	H150-1-1
Free Hemoglobin Assay Kit	Nanjing Jiancheng Bioengineering Institute	A071-1-1
TransGen All-in-One First-Strand cDNA Synthesis Kit	TransGen Biotech	AT301-02

Table S2. Primary Antibodies used in WB, IF, IHC and flow cytometry.

Application	Primary Antibodies	Dilution (v/v）	Source species	Company	Catalog No.
WB	β-actin	1:5000	Rabbit	Abcam	ab8226
WB	Tubulin	1:5000	Rabbit	Abcam	ab7291
WB	F4/80	1:1000	Rabbit	Abcam	ab300421
WB	CD68	1:1000	Rabbit	Abcam	ab283654
WB	iNOS	1:1000	Rabbit	Abcam	ab178945
WB	Arg-1	1:5000	Rabbit	Abcam	ab96183
WB	HK2	1:10000	Rabbit	Abcam	ab227198
WB	PFKP	1:500	Mouse	Abcam	ab119796

WB	PKM2	1:10000	Rabbit	Abcam	ab150377
IHC	NGAL	1:200	Rabbit	Abcam	ab125075
IF	KIM-1	1:500	Rabbit	Abcam	ab316854
Flow Cytometry	F4/80	1:100	Rat	Abcam	ab6640

Table S3. Secondary Antibodies used in WB, IF and IHC.

Secondary antibodies	Dilution (v/v）	Company	Catalog No.
HRP Goat Anti-Rabbit IgG (WB and IHC)	1:5000	Abcam	ab6721
HRP Goat Anti-Mouse IgG (WB)	1:5000	Abcam	ab6789
HRP Goat Anti-Rabbit IgG (IF)	1:400	Abcam	ab150077

Table S4. Primer sequences used for RT-qPCR.

Gene Name	Forward primer sequence (5’ to 3’)	Reverse primer sequence (5’ to 3’)
Il1b	TTCAGGCAGGCAGTATCACTC	GAAGGTCCACGGGAAAGACAC
Tnf	CTGAACTTCGGGGTGATCGG	GGCTTGTCACTCGAATTTTGAGA
Il10	AGCCTTATCGGAAATGATCCAGT	GGCCTTGTAGACACCTTGGT
Tgfb1	CTCCCGTGGCTTCTAGTGC	GCCTTAGTTTGGACAGGATCTG
Actb	GGCTGTATTCCCCTCCATCG	CCAGTTGGTAACAATGCCATGT

Figure S1 All uncropped WB membranes in this study.

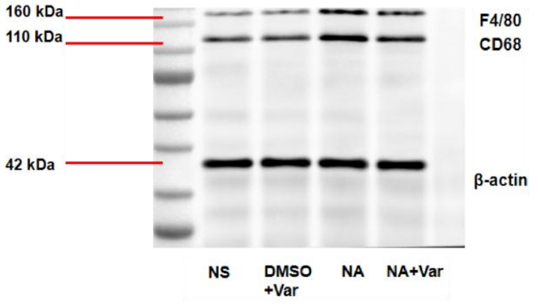

Raw WB of Fig. 2C

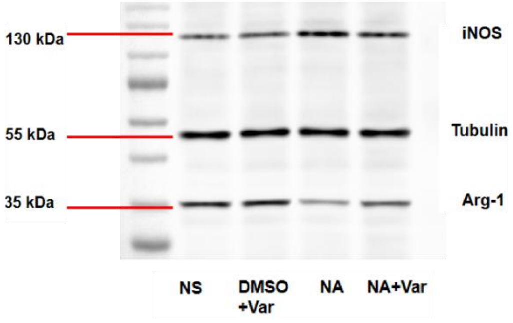

Raw WB of Fig. 3E

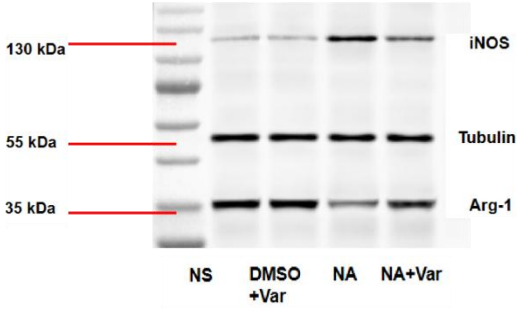

Raw WB of Fig. 4E

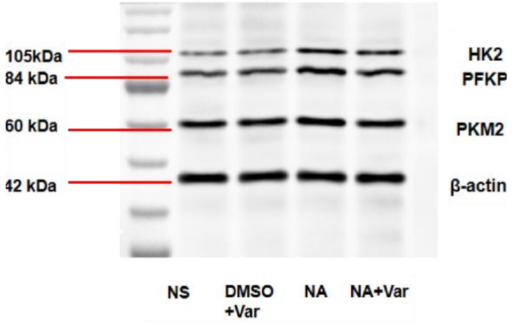

Raw WB of Fig. 5B
